# Detection of a Mammographically Occult Breast Cancer with a Challenging Clinical History

**DOI:** 10.7759/cureus.3594

**Published:** 2018-11-14

**Authors:** Quan D Nguyen, James W Randall, Taylor S Harmon, Angelica S Robinson, Claudia Cotes, Anne E Lee, Brian H Mahon, Sarfaraz Sadruddin

**Affiliations:** 1 Radiology, University of Texas Medical Branch, Galveston, USA; 2 Radiation Oncology, University of Texas Medical Branch, Galveston, USA; 3 Interventional Radiology, University of Texas Medical Branch, Galveston, USA; 4 Radiology, The Rose, Houston, USA

**Keywords:** breast cancer, invasive ductal carcinoma, mammogram, screening, bias, doppler sonography, occult breast cancer, diagnostic mammogram, guidelines, breast imaging

## Abstract

Screening mammography has helped to identify countless incidences of breast cancer since its adoption in the 1960s. Over time, the screening guidelines and techniques have been refined to better detect malignancies and to avoid false positive results. However, weaknesses remain in mammography and represent an opportunity for improvement. The interference of natural breast tissue and glands can obscure the presence of occult breast malignancies. Additionally, the inability to differentiate breast tissue on the basis of depth, and the compounding of breast densities that occurs as a consequence of two-dimensional imaging, are setbacks when it comes to relying on mammography. User error and bias can also misguide the proper detection of underlying cancers during the radiological interpretation process. The following case represents a combination of these factors and others that culminated in a missed diagnosis of invasive ductal carcinoma in a young woman suffering from mastitis of the contralateral breast.

## Introduction

Breast cancer is the most common non-skin cancer among women and the second most fatal. Recent estimates place the annual incidence of breast cancer at 266,120 in the United States, with 40,920 deaths related to the diagnosis [1]. The greatest risk for developing breast cancer occurs after the age of 70, estimated to be almost seven percent for all women above this age. Those younger than 50 typically have a very low risk of developing breast malignancy. From birth to age 49, women in the United States have close to a two percent chance of developing disease [1].

There are many risks factors associated with the development of breast cancer. One of these is the density of women's breasts. To help quantify this characteristic, there are four categories assigned by the American College of Radiology (ACR) ranging from mostly fatty tissue to extremely dense breast tissue. These are labeled 'A' to 'D' with category A being the least dense and most fatty in content. Category D is the most fibrous and the least fatty in content. While subjective, these categories are often delineated into quartiles of fibroglandular breast composition.

Dense breasts are common, especially among younger women. A large study conducted by the Breast Cancer Surveillance Consortium (BCSC) found that 43.3% of women between the ages of 40 and 74 years had heterogeneously or extremely dense breasts. The density was skewed toward younger women, with 56.6% of women between the ages of 40 and 44 years having breasts categorized as heterogeneously or extremely dense [2].

Women with breasts composed of more fibroglandular breast tissue are more likely to develop breast cancer than those of fatty composition. Those with extremely dense breasts were found to be four to six times more likely to develop breast cancer compared to those with the lowest density [3]. In addition, they are more likely to have occult disease on screening mammograms due to the masking of the fibroglandular breast tissue. One study found that women with breast imaging reporting and data system (BI-RADS) density categories of C or D were more likely to have mammographically occult disease than women with BI-RADS density categories of A or B [4]. Specifically, women with breast densities of 75% or more were almost five times more likely than women with breast densities that were less than 10% to be diagnosed with breast cancer after a negative screening examination [5]. This combination of increased risk of developing breast cancer and the likelihood of developing occult malignancy results in women with dense breasts, and are more likely to be diagnosed at a later stage of the disease [6]. To limit this, some sources suggest the addition of an ultrasound to screening mammograms in women with fibroglandular breasts in order to help detect discrete tumors at an earlier stage.  

Ultrasound is currently included in the work-up for palpable disease to help differentiate cystic from solid structures and to assess associated blood flow with doppler sonography. However, new evidence suggests the addition of sonography with screening following a negative mammogram in patients with heavily dense breasts. An Italian study of 17,883 screening mammographies observed the benefits of adding ultrasound to imaging when a suspicious mass was found [7]. A total of 6,449 screens were classified as density category C or D and were followed by ultrasound. Subsequent sonography was able to detect 29 mammographically occult cancers, accounting for 17.3% of all cancers diagnosed during the study. These may have gone undiagnosed and could have possibly presented in higher stages of malignancy or as interval cancers.

Interval cancers are defined as those which are diagnosed between screenings. Interval cancers represent false negative diagnoses, as they theoretically should not arise de novo in such a short time. For reasons already discussed, women with higher breast densities are more likely to be diagnosed with interval disease. However, this can be combated with the addition of ultrasound to the screening. An Italian retrospective cohort study was able to prove that a similar interval cancer rate can be achieved in women with category C to D breasts as those in category A or B [8]. These results suggest an improved detection of cancer in women with dense breasts when the addition of ultrasound to screening is conducted.

Another reported supplemental modality used to screen breast cancers is three-dimensional tomosynthesis. This imaging technique reduces compounding signal from overlapping tissue by using a series of low dose radiographs and digital reconstruction that produces a three-dimensional image of the breast, viewed in individual slices. Similar to typical computed tomography, the breast can be read in planes and tissues can be more accurately distinguished [9]. Regarding sensitivity and specificity, tomosynthesis plus digital mammography has already been proven to increase the area under the curve for radiologists who diagnose breast malignancy when compared to mammography alone [9]. The addition of three-dimensional tomosynthesis to mammography has demonstrated an increase in the detection of invasive and in-situ cancers, reduced false positive rates, and increased positive predictive values when compared to mammography alone [10]. Furthermore, tomosynthesis has also shown a 30% reduction in recall rates when added to mammography after initial screening [11]. When compared with mammography rather than in conjunction, three-dimensional tomosynthesis remained more efficient in reducing recall rates while maintaining a similar cancer detection rate, to conventional two-dimensional mammography [12-14]. One observational study even demonstrated improved cancer detection rates [14].

Finally, there is still controversy in the guidelines surrounding breast cancer screening. A strong debate is present over the approach to screening mammography in women between the ages of 40 to 49. The current recommendations from the ACR suggests initiating screening with mammograms at the age of 40 [15]. The American Cancer Society (ACS) argues for the benefits of annual screening beginning from between the ages of 45 to 55, then transitioning to either biennial or annual screening at the age of 55 [16]. The ACS specifically cites the more similar risk profiles of the five-year age gaps between the ages of 45 to 49 and 50 to 54, when compared to between the ages of 40 to 44. In contrast, the United States Preventive Services Task Force (USPSTF) and the World Health Organization (WHO) currently recommend biennial screening beginning at the age of 50 [17-18].

The following case describes a unique presentation of occult breast cancer in a 45-year-old woman undergoing unrelated work-up. Her situation embodies many of the arguments surrounding the current state of breast cancer screening, including proper imaging modalities, the subjective status of breast densities, physician bias, and determining the proper age to start the screening for invasive breast cancer.

## Case presentation

**Figure 1:**
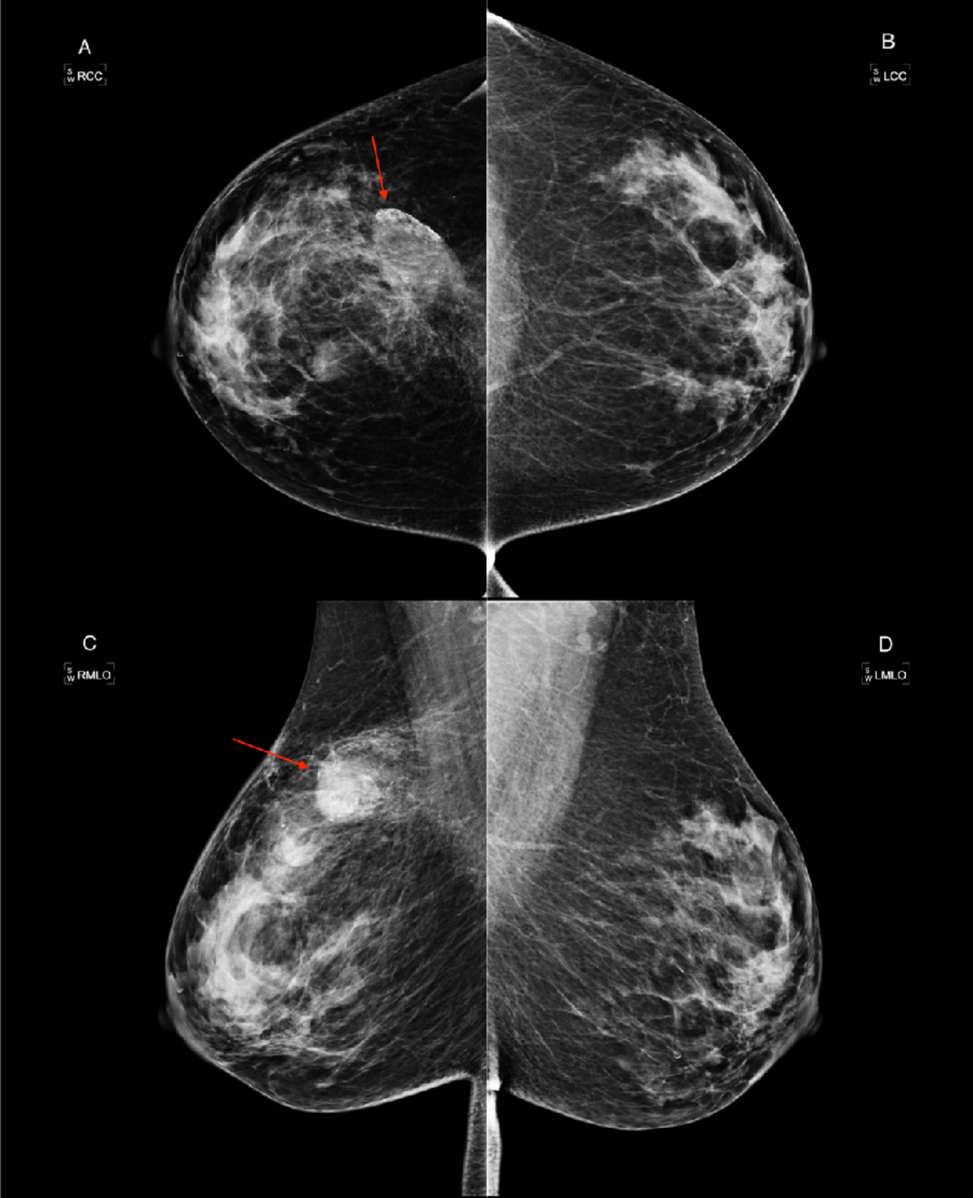
Initial Diagnostic Mammogram A diagnostic mammogram was obtained on initial presentation and shows scattered fibroglandular patterns in both breasts. The right breast includes the craniocaudal (A) and mediolateral oblique (C) views. The left breast includes craniocaudal (B) and mediolateral oblique (D) views. The demonstrated calcifications were likely caused by fat necrosis, and are observed in the right breast at the upper outer quadrant (red arrows). There are no abnormalities noted in left breast.

A 45-year-old, gravida zero para zero, female presented with a one-week history of a fluctuant mass and erythema in the right superior breast. She had a history of seat belt injury to the right breast seven years prior, and had felt stable masses in the breast for two years prior to presentation. After admission to the hospital, intravenous antibiotic therapy was initiated for symptoms of infection. No family history of breast cancer was noted at that time. The work-up for presumed mastitis began with a bilateral diagnostic mammogram. The provided patient history included a possible diagnosis of cellulitis with imaging to rule out an abscess of the right breast. The ordering physician also emphasized the history of seat belt injury. The admission diagnostic mammogram revealed heterogeneously dense breasts, as well as the presence of fat necrosis in the upper outer quadrant of the right breast at the 12 o’clock position (Figure 1).

No significant masses, calcifications, or abnormalities were noted in the left breast at that time. Ultrasound of the right breast demonstrated edema with no evidence of malignancy. The patient was diagnosed with cellulitis of the right breast and discharged with antibiotics.

**Figure 2:**
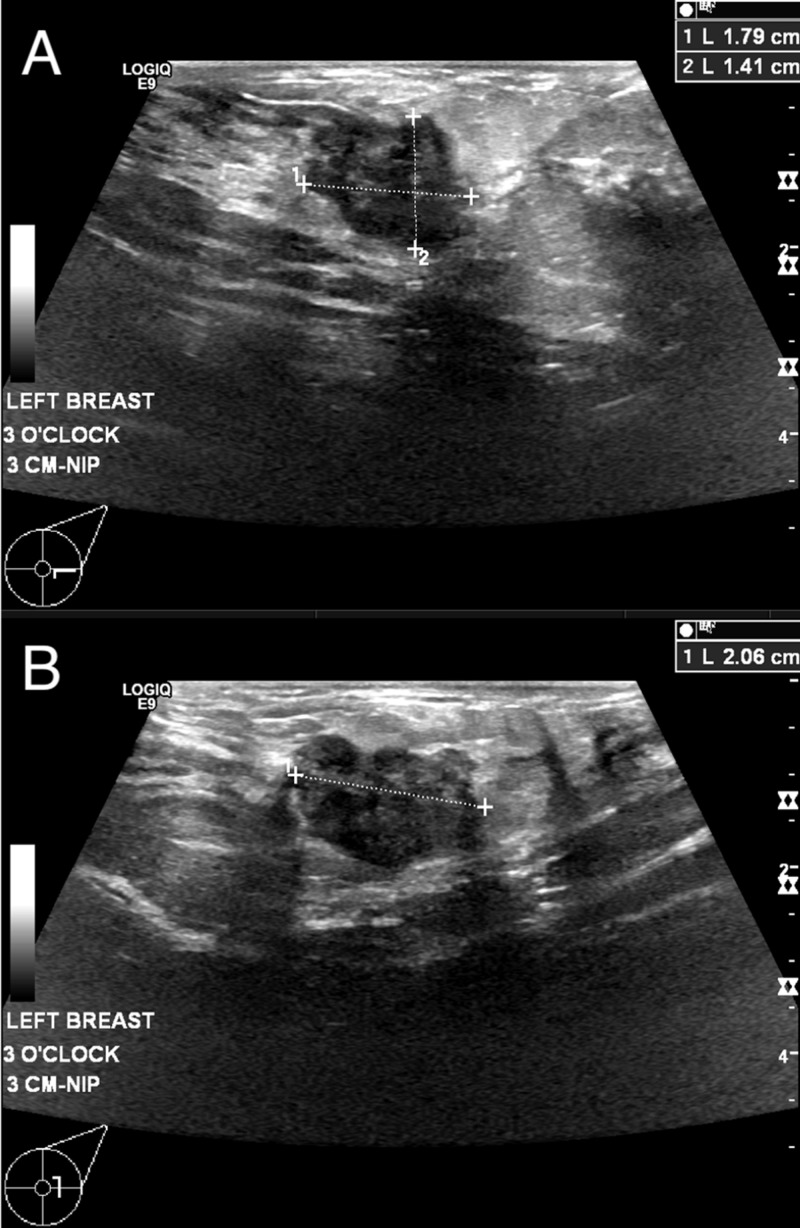
Ultrasound of the Palpable Mass An ultrasound of the left breast demonstrates overlying clinically palpable disease with the transducer in the transverse (A) and sagittal positions (B). Precise measurements can be observed in the upper right panes of the corresponding images.

Two weeks later, the same patient returned with exacerbated erythema, hardness, and tenderness in the right breast. In addition, she also noted a new lump in her left breast which she had not noticed before and mentioned this for the first time to the radiologist while ultrasound is being performed on the right side. The right breast showed redness, induration, and tenderness in the upper outer quadrant. Subsequent diagnostic ultrasound of the left breast revealed an irregularly shaped hypoechoic mass with microlobulated margins. The mass measured 21 x 18 x 14 mm and was located at the 3 o’clock position, 3 cm from the nipple (Figure 2).

Ultrasonography of the right breast revealed only fat necrosis and edema consistent with the patient history. Overall, the imaging was given a BI-RADS assessment of 4C, which is a moderate concern for malignancy.

**Figure 3:**
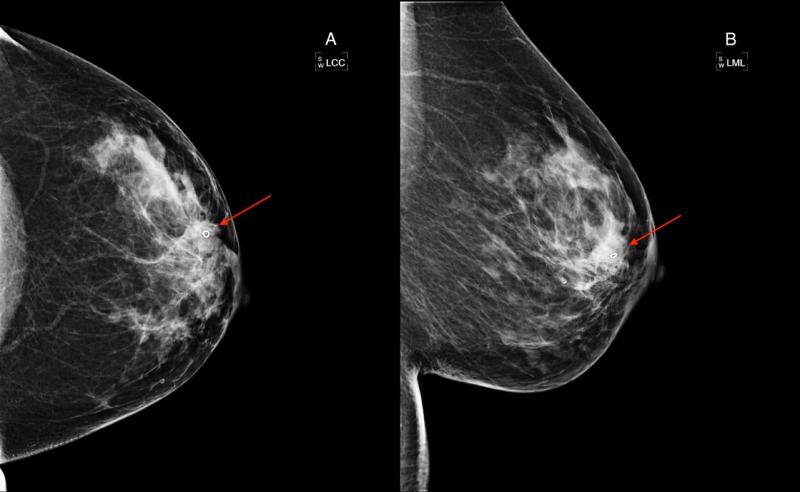
Diagnostic Mammogram following Ultrasound-guided Biopsy A diagnostic mammogram of the left breast after ultrasound guided biopsy is shown, and includes craniocaudal (A) and mediolateral (B) views. The marker indicates the biopsy site of a clinically palpable mass (red arrows). Note the unchanged fibroglandular component underlying the marker, effectively obscuring any defining features of malignancy.

Ultrasound-guided biopsy of the left breast revealed invasive ductal carcinoma, a moderately differentiated nature, and a grade of two with components of ductal carcinoma in situ. Follow-up mammography was performed, showing proper placement of a marker in the clinically observable mass (Figure 3).

The patient's care was transferred to an outside facility following a definitive diagnosis. The patient ultimately received bilateral mastectomies with sentinel lymph node dissections and adjunctive chemotherapy.

## Discussion

Many factors played a role in the false negative of the presented patient’s original diagnostic mammogram. Underlying these are the patient’s demographic information and original presentation. As a result of her age, the patient was a part of the less than two percent of women who develop breast cancer before the age of 50 [1]. When considering the patient’s age, the initial attending radiologist could have been biased to disregard any concern for occult malignancy. Additionally, it would have been possible that the radiologist's clinical intuition was deterred by the patient's infection in the contralateral breast, encouraging the neglect of the left breast and underlying malignancy. Accordingly, her history was described as, 'right breast cellulitis presenting for ultrasound to rule out abscess,' in the order for the diagnostic mammogram, predisposing the reading radiologist to focus on that area alone. Moreover, the patient had a known history of traumatic seat belt injury to her breasts and had been tracking palpable lumps for several years. Special care must be taken on behalf of both the ordering physician and the radiologist when balancing provided patient history with radiographic findings and clinical intuition.

Fortunately, this patient returned two weeks after the initial presentation for a failure of medical therapy in the contralateral breast, and received another ultrasound and diagnostic mammogram after the additional complaint of a new mass in the left breast. History of left-sided palpable breast mass was not provided by the patient to the primary provider and was only elicited after the patient discussed complaints with the attending breast imaging radiologist on her subsequent visit for contralateral disease. The radiologist who evaluated her when she came back in two weeks was not the one who did her initial diagnostic. The presented patient's case demonstrates the importance of listening to the patient and doing your own physical exam on the patient's complaints even when it differs from the reason for the exam provided by the referring provider. The thorough approach by the radiologist and not being biased by other clinical history, such as prior trauma that could cause benign palpable fat necrosis, prevented further delay in the diagnosis of the cancer.

Women with dense breast tissue are more likely to have occult disease on the screening mammogram due to masking [4]. Because of this, their disease is more likely to be diagnosed at a later stage. Mammographic density has been associated with risk of invasive tumors, tumors greater than 2.1 cm in diameter at diagnosis, and lymphatic involvement across women of all ages [6]. As expected, there is also a correlation between higher density breasts and the likelihood of undergoing mastectomy over breast-conserving therapy [4]. The patient's breasts were heterogeneously dense, obscuring the detection for masses. The patient's resulting return visit may have protected her from late-stage cancer with more invasive potential.

A delayed diagnosis in this case could have been avoided with more effective imaging. As already noted, initial three-dimensional tomosynthesis or supplementary ultrasound can be incorporated into screening guidelines to improve overall efficacy [7-14]. One concern of adding tomosynthesis to conventional mammography is increased radiation exposure. However, the increased radiation exposure is minimal in practice. Furthermore, there are computational techniques known for producing synthetic two-dimensional mammography from the three-dimensional imaging, minimizing exposure, and avoiding the need for conventional mammography altogether [19].

Following the screening protocols suggested by the USPSTF and the WHO may have left the presented patient's malignancy undiagnosed until the age of 50. At that time, the cancer may have been detected at a later, more advanced stage. The ACR and the ACS currently recommend more aggressive screening regimens when compared to other societies. These regimens are largely based on recent data that suggests there is reduced breast cancer mortality in women who undergo regular mammography from the ages of 40 and onwards [20]. The presented case represents an example of a woman who would have benefited from screening that began prior to the age of 50. In addition, these contradictory guidelines symbolize the current debate surrounding breast cancer screening and make it difficult for physicians to guide patients in these age ranges. Patients, as a result, are more likely to be confused by incongruent guidelines and could lose trust in the medical system.

Overall, the presented patient demonstrates the challenge of detecting breast cancer in dense breasts on screening mammograms. For patients with dense breasts, tomosynthesis or supplemental screening breast ultrasound may be beneficial.

## Conclusions

As currently recommended by the ACR and Society of Breast Imaging (SBI), screening for breast cancer with mammography should begin at the age of 40. For women with dense breasts, screening mammography alone could miss occult breast cancers. The addition of either screening three-dimensional tomosynthesis or supplemental breast ultrasound to screening two-dimensional mammography should be conducted to avoid false negative results. All patients with palpable breast masses and dense breasts should receive sonographic imaging. Attending radiologists should listen to the patient and do a physical exam on patient complaints on the day of the breast clinic, even when it differs from the reason for the exam provided by the referring provider.
